# Stroke-Associated Cortical Deafness: A Systematic Review of Clinical and Radiological Characteristics

**DOI:** 10.3390/brainsci11111383

**Published:** 2021-10-22

**Authors:** Gracinda Silva, Rita Gonçalves, Isabel Taveira, Maria Mouzinho, Rui Osório, Hipólito Nzwalo

**Affiliations:** 1 Faculty of Medicine and Biomedical Sciences, Campus de Gambelas, University of Algarve, 8005-139 Faro, Portugal; gracindasil@gmail.com (G.S.); mouzinho.maria@gmail.com (M.M.); ruiosorio@gmail.com (R.O.); 2 Southern Physical Medicine and Rehabilitation Center, University Hospital Center of Algarve, 8150-022 São Brás de Alportel, Portugal; ritamg8@gmail.com; 3 Intensive Care Unit, University Hospital Center of Algarve, Rua Leão Penedo, 8000-386 Faro, Portugal; isabeltaveira89@gmail.com; 4 Stroke Unit, University Hospital Center of Algarve, Rua Leão Penedo, 8000-386 Faro, Portugal

**Keywords:** cortical deafness, stroke, auditory agnosia

## Abstract

Background: Stroke is the leading cause of cortical deafness (CD), the most severe form of central hearing impairment. CD remains poorly characterized and perhaps underdiagnosed. We perform a systematic review to describe the clinical and radiological features of stroke-associated CD. Methods: PubMed and the Web of Science databases were used to identify relevant publications up to 30 June 2021 using the MeSH terms: “deafness” and “stroke”, or “hearing loss” and “stroke” or “auditory agnosia” and “stroke”. Results: We found 46 cases, caused by bilateral lesions within the central auditory pathway, mostly located within or surrounding the superior temporal lobe gyri and/or the Heschl’s gyri (30/81%). In five (13.51%) patients, CD was caused by the subcortical hemispheric and in two (0.05%) in brainstem lesions. Sensorineural hearing loss was universal. Occasionally, a misdiagnosis by peripheral or psychiatric disorders occurred. A few (20%) had clinical improvement, with a regained oral conversation or evolution to pure word deafness (36.6%). A persistent inability of oral communication occurred in 43.3%. A full recovery of conversation was restricted to patients with subcortical lesions. Conclusions: Stroke-associated CD is rare, severe and results from combinations of cortical and subcortical lesions within the central auditory pathway. The recovery of functional hearing occurs, essentially, when caused by subcortical lesions.

## 1. Introduction

Central hearing impairment (CHI) encompasses a continuum of auditory disorders resulting from lesions within the central nervous system [1]. The clinical spectrum of CHI includes cortical deafness (CD), word sound deafness, word meaning deafness, nonverbal auditory agnosia and receptive amusia [1,2,3,4,5]. Stroke is a leading cause of acquired CHI [2,3,4]. CD, the severest manifestation of CHI, is characterized by the loss of the ability to perceive auditory signals by the cortex, despite normal peripheral hearing [2]. Patients with CD retain the ability to speak, read, write and, occasionally, react to very intense sound levels [2,3,4]. This complication can be transient or even progress to a less severe manifestation of CHI [2,6]. Data on stroke-associated CD are extremely sparse and come from descriptions of isolated published cases. For this reason, prompt recognition, clinical management and prognostication are certainly problematic. Previous reviews on the topic are narrative, based on highly selective groups, for instance, cases of persistent CD or included mixed stroke and non-stroke populations [2,6,7]. Therefore, we decided to perform a systematic review centered specifically on stroke-associated CD.

## 2. Materials and Methods

We used PubMed and Scopus databases to search for relevant publications from inception up to 30 June 2021 using a combination of the following MeSH terms: “deafness” and “stroke”, or “hearing loss” and “stroke” or “auditory agnosia” and “stroke”. This search was complemented by examining reference lists of the most relevant publications. Manuscripts describing cases of stroke-associated acquired loss of understanding verbal and non-verbal sounds were considered eligible. Two co-authors (speech therapists) reviewed each manuscript to certify that the patients described fulfilled the criteria for CD. Cases of non-stroke-related CD (traumatic, tumoral, etc.), other types of CHI and non-English language publications were excluded. In addition, we excluded cases of deafness resulting from vascular lesions affecting the peripheral auditory system (cochlea, vestibulocochlear nerve). For each included case, the following data were extracted: sociodemographics (age, gender), clinico-radiological characteristics (stroke main type, location of the lesions, timing of the strokes, clinical evolution of CD, speech therapy), evidence of sensorineural hearing loss (pure tone audiometry), brainstem auditory evoked potentials (evidence of integrity of peripheral central nervous auditory pathway). The World Health Organization (WHO) classification of hearing impairment was adapted to classify the sensorineural hearing loss (https://www.who.int/pbd/deafness/hearing_impairment_grades/en/) (accessed on 15 May 2021): mild ≤ 40 dB, moderate 41–60 dB and severe > 60 dB. All obtained titles and abstracts were independently verified by 2 investigators. Disagreements regarding the inclusion of specific studies were resolved by a third investigator.

## 3. Results

### Data Collection

**Figure 1:**
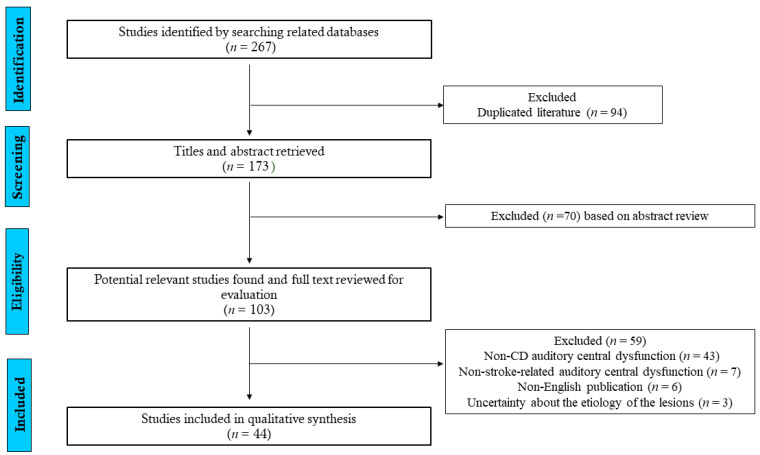
Prisma flowchart process of selection and inclusion of articles in the systematic review.

A total of 267 references was initially retrieved. After the automatic removal of duplicated manuscripts, 173 articles were screened. A Preferred Reporting Items for Systematic Reviews and Meta-Analyses (PRISMA) flowchart diagram (Figure 1) resumed the selection and inclusion process.

A total of 103 manuscripts was selected for a complete text evaluation, after which 44 were included [2,4,7,8,9,10,11,12,13,14,15,16,17,18,19,20,21,22,23,24,25,26,27,28,29,30,31,32,33,34,35,36,37,38,39,40,41,42,43,44,45,46,47,48]. The total number of patients included was 46 (Table 1).

**Figure 2:**
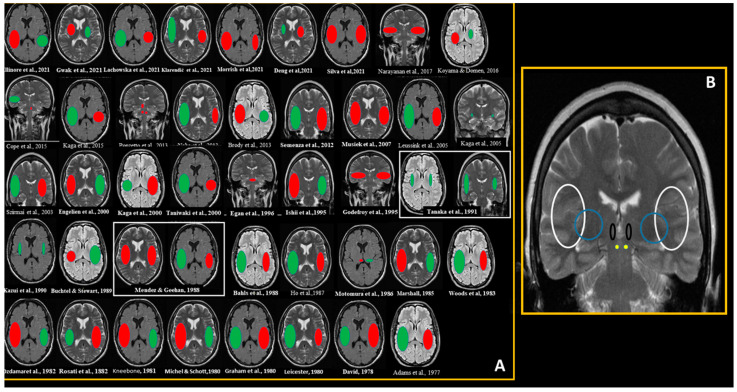
Approximate lesion location in cases of stroke-associated cortical deafness. In (**A**), there was an attempt to replicate the location from each case included in the systematic review. Red represents acute stroke and green, subacute or chronic stroke. Size asymmetries represent hemispheric differences in the size of stroke lesions. In (**B**), there was a representation of topographic locations of lesions associated with cortical deafness (white semicircles for temporal lobe, blue circles for basal ganglia/auditory radiations, black semicircles for thalamus and yellow dots for inferior colliculi).

The main reasons for exclusion (*n* = 59) were non-CD manifestations of CHI (*n* = 43) and non-stroke-related auditory central dysfunction (*n* = 7) (Table S1). The mean age was 51.4 years (range 21–82 years), the majority were males (28/60.8%) and ischemic stroke was the most common subtype (30/65.2%). With rare exceptions [20,25,31,32], CD resulted from lesions affecting the hemispheres bilaterally. In a minority of patients with hemispheric stroke [13,17,21,26,31,34,37], lesions were not in or near the superior temporal gyrus or Heschl’s gyri (6/15%). Figure 2 shows that lesions were located within or in the vicinity structures that are part of the central auditory pathway. In these cases, effective disconnection from lesions disrupting the auditory pathway, in the basal ganglia, internal capsule, inferior colliculus, thalamic regions and medial geniculate body were implicated. In the majority of cases caused by bilateral hemispheric stroke, the large lesion was right-sided.

The brainstem auditory-evoked potentials were normal in all cases. With a few exceptions [24,32,45], the results of pure tone audiometry were documented. Table 1 demonstrates that moderate to severe sensorineural hearing loss was documented in all. In a large group of cases (*n* = 21), no information about speech therapy was available. The duration of the follow-up varied from 2 weeks to 15 years. A substantial part of the patients did not improve, and remained with a persistent inability to recognize any sound at all (16/34.8%) [2,8,12,15,16,17,18,19,29,33,34,35,37,43,46,48], while others evolved to pure word deafness (11/36.6%) [7,9,10,11,13,18,21,22,24,31,36,39,41,42]. Oral conversation was regained in six (20%) of the patients [7,20,25,27,28,32,42,43]. In two patients, CD was transient, one following a bilateral middle cerebral vasospasm secondary to an aneurysm rupture [30] and the other two after a unilateral ischemic stroke [38,40].

## 4. Discussion

This systematic review confirmed the extreme rarity of stroke-associated CD. However, because alternative diagnoses, such as peripheral hearing disease or even psychiatric disorder [2,29,36], were reported, the possibility of a misdiagnosis should be considered. The coexistence of CD with language impairment, in particular with Wernicke’s aphasia, is a reality [49] and may further complicate prompt recognition. Therefore, in patients with lesions involving the central auditory pathway, whether cortical or subcortical, simultaneous or not, the exclusion of CD and other CHI should be active. The understanding and correct interpretation of sounds within the environment depends on the integrity of the ears as well of specific brain regions such as the cochlear nuclei, superior olivary nuclei, lateral lemniscus, inferior colliculus, medial geniculate nuclei and auditory cortex [50]. CD is essentially thought to result from bilateral lesions of the primary auditory cortex located in the temporal lobes [2,3,4]. This systematic review demonstrated that combinations of bilateral stroke lesions in the brainstem [20,25,32], subcortical hemispheric [13,21,23,24,26,31,34] or cortical with contralateral subcortical hemispheric [9,24] can cause CD (Figure 2). The auditory input from each of the ears travels along bilateral subcortical connections to both auditory cortices, where sound is interpreted [50,51]. The redundancy of an auditory cortical representation is the reason why CD is seldom reported after stroke [51]. In other words, a combination of strategic bilateral injury of structures within the auditory pathway from the brainstem to the temporal lobes is a sine qua non condition to stroke-associated CD. Notably, more than a third of patients with post-stroke CD evolved to a less severe CHI, namely, pure word deafness or auditory verbal agnosia, which is the inability to comprehend speech with a preserved comprehension of non-verbal sounds [5]. With a few exceptions, all of them resulting from transient ischemia [30,40], some level of auditory dysfunction persisted. Remarkably, only in cases of CD caused by the interruption of subcortical acoustic radiations [20,25] or by a secondary auditory area [43], a marked improvement leading to functional oral communication was observed on the follow-up. This was consistent with the findings from other cortical deficits caused by interruptions of cortico-subcortical circuits, for instance in patients with subcortical aphasia [52]. In subcortical aphasia, recovering is in general better and faster [52]. This improvement is boosted by an increased intrahemispheric functional connectivity and decreased interhemispheric functional connectivity [53], a combination of mechanisms associated with better outcomes in patients recovering from stroke [54]. Therapy-induced plasticity plays a central role in enhancing functional connectivity and, by that, in improving recovery [54]. However, in stroke patients with CD, the patient is deaf; therefore, “inaccessible to rehabilitation”. Indeed, the poor recovery of patients with post-stroke CD somehow resembles the evolution of patients with post stroke cortical blindness, which are also “inaccessible to rehabilitation” [55]. There is some evidence showing that focus training on attention abilities towards the awareness of sounds may improve the comprehension of sounds [23]. Anecdotal cases have shown that transcranial direct current stimulation can improve verbal comprehension in patients with auditory agnosia [56]. Because deafness prevents the effectiveness of therapy-induced plasticity, direct current stimulation could emerge as a potential strategy of stroke-associated CD. Another possible intervention is personal frequency-modulated systems, which have been shown to improve speech in noise perception in patients with stroke with CHI, probably through the improvement of auditory neuroplasticity [57]. One of the physiological substrates of personal frequency-modulated systems is the delivery of an intense input level of speech to the patients [57]. Hence, at least in patients retaining residual hearing who respond to intense auditory stimuli, this intervention can potentially enhance auditory neuroplasticity and improve prognosis. There were very important limitations in our systematic review. The number of cases was sparse and the duration of the follow-up was variable. In addition, very often, patients with CD also expressed other cortical dysfunctions that could have complicated the diagnosis and interpretation of the prognosis. For these reasons, the robustness of our conclusions is limited.

## 5. Conclusions

Our systematic review showed that stroke-associated CD is a severe condition, and occurs in patients with bilateral cortical and subcortical lesions within the central auditory pathway. Misdiagnosis is a possibility. Prognosis is better when caused by subcortical lesions. Improvement is a possibility, often with an evolution to pure word deafness, a less severe form of CHI.

## Figures and Tables

**Table 1:** Sociodemographics, clinical and radiological characteristics of the 46 cases of stroke-associated cortical deafness included in the systematic review.

Author, Year	Age, Gender	Stroke Type	Topography	SHL	SR	Evolution
(Ellinore et al., 2021) [38]	53, F	I	Bilateral: STG, Heschl’s gyri	Mild	Yes	Improvement (4 h): total recovery
(Gwak et al., 2021) [43]	41, F	H	Bilateral: basal ganglia	Severe	Yes	Persistence CD (6 months)
(Lachowska et al., 2021) [41]	46, F	I	R: STG, Heschl’s gyri L: Heschl’s gyri	Severe	Yes	Improvement (10 months): pure word deafness
(Klarendić et al., 2021) [42]	66, F	I	R: frontal lobe, Insula L: STG, Heschl’s gyri	Severe	Yes	Improvement (7 days): oral conversation possible
(Morrish et al., 2021) [40]	65, M	I	Bilateral: STG, Heschl’s gyri	No data	Not applicable	Improvement (after thrombectomy): total recovery
(Deng et al., 2020) [37]	50, M	H, I	R: basal ganglia L: paraventricular	No data	No data	Persistence CD (6 months)
(Silva et al., 2020) [36]	32, F	I	Bilateral: STG, Heschl’s gyri	Moderate	Yes	Improvement (3 months): pure word deafness
(Narayanan et al., 2017) [35]	58, M	I	Bilateral STG, Heschl’s gyri	Severe	Yes	Persistence CD (unknown follow-up duration)
(Koyama and Domen, 2016) [34]	59, F	H	Bilateral globus pallidus, internal capsule	Severe	Yes	Persistence CD (24 months)
(Cope et al., 2015) [32]	48, M	H	R: extensive temporal lobe L: internal colliculus	No data	Yes	Improvement (36 months): oral conversation possible
(Kaga et al., 2015) [33]	38, M	I*	Bilateral: STG, Heschl’s gyri, auditory radiations	Severe	Yes	Persistence CD (36 months)
(Ponzetto et al., 2013) [31]	55, F	I*	R: hippocampus, thalamus Bilateral: pons, periaqueductal vicinity	Moderate	Yes	Improvement (6 months): pure word deafness
(Ramdasi and Chagla, 2014) [30]	32, M	I*	No lesions (bilateral vasospasm of the middle cerebral artery)	Moderate	Not applicable	Improvement (5 days): total recovery
(Naha et al., 2013) [29]	49, M	I	Bilateral: STG, Heschl’s gyri, MTG, insula	Mild	No data	Persistence CD (unknown follow-up duration)
(Brody et al., 2013) [9]	56, F	H	R:STG, Heschl’s gyri, insula L: thalamus, globus pallidus, internal and external capsule	Severe	Yes	Improvement (36 months): pure word deafness
(Semenza et al., 2012) [8]	55, F	I	Bilateral: STG, Heschl’s gyri, insula, angular gyrus, supramarginal gyrus	Severe	Yes	Persistence CD (36 month)
(Musiek et al., 2007) [28]	46, F	I	Bilateral: STG, Heschl’s gyri, MTG, insula	Severe	Yes	Improvement (24 months): communication using combined oral, written and non-verbal language
(Leussink et al., 2005) [27]	74, F	I	R: STG, Heschl’s gyri, MTG, insula L: Heschl’s gyri	Severe	Yes	Improvement (2 weeks): recovering of perception of words
(Kaga et al., 2005) [26]	43, M	H	Bilateral: putamen, bilateral auditory radiations	Mild	No data	No data
(Musiek et al., 2004) [25]	21, M	H	Bilateral inferior colliculi	Moderate	Yes	Improvement (12 months): able to follow most conversations
(Szirmai et al., 2003) [24]	58, M	H	R: striatum, internal capsule, L: STG, insula, supramarginal gyrus	No data	Yes	Improvement (6 months): pure word deafness
(Engelien et al., 2000) [23]	22, M	I	Bilateral: STG, Heschl’s gyri, insula	Normal	Yes	No data
(Kaga et al., 2000) [22]	37, M	I	Bilateral: Heschl’s gyrus, medial geniculate body	Moderate	Yes	Mild improvement (>36 months): poor recognition of speech
(Taniwaki et al., 2000) [21]	46, F	H	Bilateral: putamen, bilateral auditory radiation	Severe	No data	Improvement (1 month): pure word deafness
(Egan et al., 1996) [20]	64, F	H	Midline pontine tegmentum	Moderate	No data	Improvement (1 month): significant language comprehension
(Ishii, Kazuhiro Ueda et al., 1995) [19]	55, M	I	Bilateral STG, Heschl’s gyri	Moderate	No data	Persistence CD (24 months)
(Godefroy et al., 1995) [18]	58, M	H	Bilateral STG, external capsule	Severe	Yes	Improvement (2 months): pure word deafness
(Tanaka et al., 1991) [17]	48, M	H	Bilateral putamen, insula	Severe	No data	Persistence CD (4 months)
(Tanaka et al., 1991) [17]	38, M	I	Bilateral STG, Heschl’s gyri, insula	Severe	No data	Persistence CD (6 months)
(Kazui et al., 1990) [16]	66, M	I	R: temporal stem, insula, Heschl’s gyrus. L: parietal, temporal stem.	Severe	No data	Persistence of CD (7 months)
(Buchtel and Stewart, 1989) [4]	51, M	I	L: frontotemporal, parieto-temporal R: posterior temporal	Moderate	No data	No data
(Mendez and Geehan, 1988) [7]	60, M	H	Bilateral STG	Severe	No data	Improvement (2 weeks): pure word deafness
(Mendez and Geehan, 1988) [7]	23, M	H	R: fronto-parietotemporal L: parietotemporal	Mild	No data	Improvement (7 months): significant language comprehension
(Fredrick et al., 1988) [19]	61, M	I	Bilateral STG, Heschl’s gyri	Mild	Yes	Persistence of CD (2 years)
(Ho et al., 1987) [14]	67, F	I	R: supramarginal, angular gyri L: STG, Heschl’s gyri	Mild	No data	No data
(Motomura et al., 1986) [13]	69, M	I, H	L: thalamus, Internal capsule R: internal capsule	Mild	Yes	Improvement (2 months): pure word deafness
(Marshall, 1985) [12]	62, F	I	R: STG, MTG, Heschl’s gyrus L: STG, geniculotemporal tract, insula	Mild	Yes	Persistence CD (36 months)
(Woods et al., 1984) [11]	82, F	I	Bilateral STG, MTG, Heschl’s gyrus	Moderate	Yes	Improvement (3 months): pure word deafness
(Ozcan et al., 1982) [10]	36, F	I*, H	Bilateral STG, MTG, insula	Severe	No data	Improvement (17 months): pure word deafness
(Rosati et al., 1982) [48]	49, M	I	Bilateral STG, MTG	Mild	No data	Persistence of CD (7 months)
(Kneebone CS, 1981) [39]	70, M	I	Bilateral extensive temporal lobe	Severe	Yes	Improvement (12 months): pure word deafness
(Michel and Schott, 1980) [47]	40, M	I	Bilateral STG, MTG	Severe	No data	No data
(Graham et al., 1980) [2]	48, F	I	Bilateral extensive temporal lobe	Severe	No data	Persistence of CD (6 months)
(Leicester, 1980) [46]	62, M	I	Bilateral STG, MTG, Heschl’s gyrus	Severe	No data	Persistence of CD (24 months)
(David, 1978) [45]	64, M	I	Bilateral STG, Heschl’s gyri	No data	No data	No data
(Adams et al., 1977) [44]	42, M	I	Bilateral extensive temporal lobe	Severe	No data	No data

I*: vasospasm after aneurysmal subarachnoid hemorrhage; I: ischemic; H: hemorrhagic; R: right; L: left; STG: superior temporal gyrus; MTG: middle temporal gyrus; SR: speech rehabilitation; SHL: sensorineural hearing loss.

## Supplementary Materials

The following are available online at https://www.mdpi.com/article/10.3390/brainsci11111383/s1, Table S1: list and causes of exclusion from the systematic review.
