# Supplementary material for: Stroke-Associated Cortical Deafness: A Systematic Review of Clinical and Radiological Characteristics
Source: Brain Sci. 2021 Oct 22;11(11):1383. doi: 10.3390/brainsci11111383 (PMC8615419; doi:10.3390/brainsci11111383)
Supplement: Supplementary file 1 [file brainsci-11-01383-s001.zip › brainsci-1420522-supplementary.pdf]

Supplemental file: List of excluded articles

Reference	Reason
**Kinouchi T, Ishitani K, Uyama S, Miyamoto T, Fujimoto N, Ueta H. Basilar artery occlusion presenting as sudden bilateral deafness: a case report. J Med Case Rep. 2021 Mar 2;15(1):111.**	Non-CD auditory central dysfunction
**Slotwinski K, Ejma M, Szczepanska A, Budrewicz S, Koszewicz M. Pure word deafness in a patient with bilateral ischemic stroke in the superior temporal gyrus (STG). Neurocase. 2020 Jun;26(3):121-124.**	Non-CD auditory central dysfunction
**Ben Younes T, Messelmani M, Mansour M, Zaouali J, Mrissa R. Pure word deafness revealing ischemic stroke in a Tunisian patient. Clin Neurol Neurosurg. 2019 Dec;187:105541.**	Non-CD auditory central dysfunction
**Shibata Y. Delayed traumatic intracerebral hematoma presenting as cortical deafness: case report. Heliyon. 2020;6(2):e03443.**	Non-stroke related auditory central dysfunction
**Kim JM, Woo SB, Lee Z, Heo SJ, Park D. Verbal auditory agnosia in a patient with traumatic brain injury: A case report. Medicine (Baltimore). 2018 Mar;97(11):e0136.**	Non-stroke related auditory central dysfunction
**Sugiura T, Torii T. Auditory agnosia caused by bilateral putamen haemorrhage. BMJ Case Rep. 2017 Dec 20;2017:bcr2017222535.**	Non-CD auditory central dysfunction
**Maffei C, Capasso R, Cazzolli G, Colosimo C, Dell'Acqua F, Piludu F, Catani M, Miceli G. Pure word deafness following left temporal damage: Behavioral and neuroanatomical evidence from a new case. Cortex. 2017;97:240–54.**	Non-CD auditory central dysfunction

**Poliva O, Bestelmeyer PEG, Hall M, Bultitude JH, Koller K, Rafal RD. Functional mapping of the human auditory cortex: FMRI investigation of a patient with auditory agnosia from trauma to the inferior colliculus. Cogn Behav Neurol. 2015;28(3):160–80.**	Non-stroke related auditory central dysfunction
**Gutschalk, A., Uppenkamp, S., Riedel, B., Bartsch, A., Brandt, T., & Vogt-schaden, M. (2015). ScienceDirect Pure word deafness with auditory object agnosia after bilateral lesion of the superior temporal sulcus. Cortex. *73*, 24–35**	Non-CD auditory central dysfunction
**Joswig H, Schönenberger U, Brügge D, Richter H, Surbeck W. Reversible pure word deafness due to inferior colliculi compression by a pineal germinoma in a young adult. Clin Neurol Neurosurg. 2015;139:62–5.**	Non-stroke related auditory central dysfunction
**Cavinato M, Rigon J, Volpato C, Semenza C, Piccione F. Preservation of Auditory P300-Like Potentials in Cortical Deafness. PLoS One. 2012;7(1):1-6.**	Data presented by Semenza et al, 2012 **–** Semenza C, Cavinato M, Rigon J, Battel I, Meneghello F, Venneri A. Persistent cortical deafness: A voxel-based morphometry and tractography study. Neuropsychology. 2012;26(6):675–83.
**Chiang CI, Chou CH, Hsueh CJ, Cheng CA, Peng GS. Acute bilateral hearing loss as a "worsening sign" in a patient with critical basilar artery stenosis. J Clin Neurosci. 2013 Jan;20(1):177-9.**	Non-CD auditory central dysfunction

**Saygin AP, Leech R, Dick F. Nonverbal auditory agnosia with lesion to Wernicke's area. Neuropsychologia. 2010;48(1):107–13.**	Non-CD auditory central dysfunction
**Mun SK, Hong YH, Kang SH, Hwang SN. A case of temporal intracerebral hemorrhage that presented with sudden bilateral hearing loss as the initial symptom. J Korean Neurosurg Soc. 2010 Nov;48(5):438-40.**	Non-CD auditory central dysfunction
**Gerace C, Pianura C. Sudden deafness without vertigo as a sole manifestation of AICA infarct. Neurol Sci. 2008 Oct;29(5):371-2.**	Non-CD auditory central dysfunction
**Bovo R, Ortore R, Ciorba A, Berto A, Martini A. Bilateral sudden profound hearing loss and vertigo as a unique manifestation of bilateral symmetric inferior pontine infarctions. Ann Otol Rhinol Laryngol. 2007 Jun;116(6):407-10.**	Non-CD auditory central dysfunction
**Sugiura T, Torii T. Auditory agnosia caused by bilateral putamen haemorrhage. *BMJ Case Rep*. 2017;2017:bcr2017222535.**	Non-CD auditory central dysfunction
**Tabuchi S, Kadowaki M, Watanabe T. Reversible cortical auditory dysfunction caused by cerebral vasospasm after ruptured aneurysmal subarachnoid hemorrhage and evaluated by perfusion magnetic resonance imaging. Case report. J Neurosurg. 2007 Jul;107(1):161-4.**	Non-CD auditory central dysfunction
**Satoh M, Takeda K, Kuzuhara S. A case of auditory agnosia with impairment of perception and expression of music: Cognitive processing of tonality. Eur Neurol. 2007;58(2):70–7.**	Non-CD auditory central dysfunction
**Plasencia PM, Dorado JI, Serrano Rodríguez JM, Sellán C. Neuropsychological evidence for "word-meaning deafness" in a Spanish-speaking patient. Brain Lang. 2006 May;97(2):214-8.**	Non-CD auditory central dysfunction

**Yi HA, Lee SR, Lee H, Ahn BH, Park BR, Whitman GT. Sudden deafness as a sign of stroke with normal diffusion-weighted brain MRI. Acta Otolaryngol. 2005 Oct;125(10):1119-21.**	Non-CD auditory central dysfunction
**Kimiskidis VK, Lalaki P, Papagiannopoulos S, Tsitouridis I, Tolika T, Serasli E, Kazis D, Tsara V, Tsalighopoulos MG, Kazis A. Sensorineural hearing loss and word deafness caused by a mesencephalic lesion: Clinicoelectrophysiologic correlations. Otol Neurotol. 2004;25(2):178–82.**	Non-CD auditory central dysfunction
**Fernández CA, Carceller MA, García JR, García CG, Alegría JB. Sudden deafness as a manifestation of the rupture of a cerebral arteriovenous malformation. Otolaryngol Head Neck Surg. 2003 Apr;128(4):592-4.**	Non-CD auditory central dysfunction
**Vitte E, Tankéré F, Bernat I, Zouaoui A, Lamas G, Soudant J. Midbrain deafness with normal brainstem auditory evoked potentials. Neurology. 2002;58(6):970–3.**	Non-stroke related auditory central dysfunction
**Sato M, Yasui N, Isobe I, Kobayashi T. [A case of pure word deafness and auditory agnosia associated with bilateral temporo-parietal lesions]. No To Shinkei. 1982 Oct;34(10):939-45. Japanese.**	Non-English language
**Akkuzu B, Fişiloğlu AG, Ozlüoğlu L, Can U. Sudden cortical hearing loss for speech: a case report. Ear Hear. 2001 Feb;22(1):14-7.**	Non-CD auditory central dysfunction
**Mendez MF. Generalized auditory agnosia with spared music recognition in a left-hander. Analysis of a case with a right temporal stroke. Cortex. 2001;37(1):139–50.**	Non-CD auditory central dysfunction
**Yaguchi H, Yaguchi M, Nishiwaki C, Takahashi Y. [A case of brain stem infarction with bilateral hearing loss]. No To Shinkei. 2000 Mar;52(3):249-52. Japanese.**	Non-English language

**Nakayama T, Nobuoka H, Wada S, Matsukado Y. Cortical deafness following bilateral hypertensive putaminal hemorrhage. No To Shinkei. 1986;38:565–570.**	Non-English language
**Clarke S, Bellmann A, Meuli RA, Assal G, Steck AJ. Auditory agnosia and auditory spatial deficits following left hemispheric lesions: evidence for distinct processing pathways. Neuropsychologia. 2000;38(6):797-807.**	Non-CD auditory central dysfunction
**Wang E, Peach RK, Xu Y, Schneck M, Manry II C. Perception of dynamic acoustic patterns by an individual with unilateral verbal auditory agnosia. Brain Lang. 2000;73(3):442–55.**	Non-CD auditory central dysfunction
**Bhaskaran R, Prakash M, Kumar PN, Srikumar B. Crossed aphasia leading to pure word deafness. J Assoc Physicians India. 1998 Sep;46(9):824-6.**	Non-CD auditory central dysfunction
**Johkura K, Matsumoto S, Hasegawa O, Kuroiwa Y. Defective auditory recognition after small hemorrhage in the inferior colliculi. J Neurol Sci. 1998;161(1):91–6.**	Non-stroke related auditory central dysfunction
**Johannes S, Jöbges ME, Dengler R, Münte TF. Cortical auditory disorders: a case of non-verbal disturbances assessed with event-related brain potentials. Behav Neurol. 1998;11(1):55-73.**	Non-CD auditory central dysfunction
**Deplanque D, Godefroy O, Guerouaou D, Laureau E, Desaulty A. Sudden bilateral deafness: lateral inferior pontine infarction. J Neurol Neurosurg Psychiatry. 1998 Jun;64(6):817-8.**	Non-CD auditory central dysfunction
**Papathanasiou I, Macfarlane S, Heron C. A Case of Verbal Auditory Agnosia: Missing the Word…Missing the Sound…. Int J Lang Commun Disord. 1998;33(S1):214–7.**	Non-CD auditory central dysfunction
**Hu C-J, Chan K-Y, Lin T-J, Hsiao S-H, Chang Y-M, Sung S-M. Traumatic brainstem deafness with normal brainstem auditory evoked potentials. Neurology. 1997 May 1;48(5):1448–51.**	Non-stroke related auditory central dysfunction

**Habib M, Daquin G, Milandre L, Royere ML, Rey M, Lanteri A, Salamon G, Khalil R. Mutism and auditory agnosia due to bilateral insular damage-Role of the insula in human communication. Neuropsychologia. 1995;33(3):327–39.**	Non-CD auditory central dysfunction
**Di Giovanni M, D'Alessandro G, Baldini S, Cantalupi D, Bottacchi E. Clinical and neuroradiological findings in a case of pure word deafness. Ital J Neurol Sci. 1992;13(6):507–10.**	Non-CD auditory central dysfunction
**Praamstra P, Hagoort P, Maassen B, Crul T. Word deafness and auditory cortical function. A case history and hypothesis. Brain. 1991 Jun 1;114 (Pt 3(3):1197–225.**	Non-CD auditory central dysfunction
**Shindo M, Kaga K, Tanaka Y. Speech discrimination and lip reading in patients with word deafness or auditory agnosia. Brain Lang. 1991;40(2):153–61.**	Non-CD auditory central dysfunction
**Wolberg SC, Temlett JA, Fritz VU. Pure word deafness. S Afr Med J. 1990 Dec 1;78(11):668-70.**	Non-CD auditory central dysfunction
**Fujii T, Fukatsu R, Watabe S ichi, Ohnuma A, Teramura K, Kimura I, Saso S, Kogure, K. Auditory Sound Agnosia without Aphasia Following a Right Temporal Lobe Lesion. Cortex. 1990;26(2):263–8.**	Non-CD auditory central dysfunction
**Bales JD. Reversible sensorineural hearing loss in a stroke patient. Ear Hear. 1989 Apr;10(2):109-11.**	Non-CD auditory central dysfunction
**Yaqub BA, Gascon GG, Nosha M Al, Whitaker H. Pure word deafness (acquired verbal auditory agnosia) in an arabic speaking patient. Brain. 1988;111(2):457–66.**	Non-CD auditory central dysfunction
**Roberts M, Sandercock P, Ghadiali E. Pure word deafness and unilateral right temporo-parietal lesions: a case report. J Neurol Neurosurg Psychiatry. 1987 Dec;50(12):1708-9.**	Non-CD auditory central dysfunction

**Meissner R. Doppelseitige zentrale Hörstörung infolge bitemporalen Hirninfarktes [Double-sided central hearing-loss owing to bitemporal infarct of brain (author's transl)]. HNO. 1981 Aug;29(8):282-4. German.**	Non-English language
**Buchman AS, Garron DC, Trost-Cardamone JE, Wichter MD, Schwartz M. Word deafness: one hundred years later. J Neurol Neurosurg Psychiatry. 1986 May;49(5):489-99.**	Non-CD auditory central dysfunction
**Coslett HB, Brashear HR, Heilman KM. Pure word deafness after bilateral primary auditory cortex infarcts. Neurology. 1984 Mar;34(3):347-52.**	Non-CD auditory central dysfunction
**Pamphlett R, Morris J. Cortical hearing deficit. A deaf brain. Med J Aust. 1983;2(1):35–6.**	Non-CD auditory central dysfunction
**Auerbach SH, Allard T, Naeser M, Alexander MP, Albert ML. Pure word deafness: Analysis of a case with bilateral lesions and a defect at the prephonemic level. Brain. 1982;105(2):271–300.**	Non-CD auditory central dysfunction
**Khurana RK, O'Donnell PP, Suter CM, Inayatullah M. Bilateral deafness of vascular origin. Stroke. 1981 Jul-Aug;12(4):521-3.**	Non-CD auditory central dysfunction
**Tsuruoka H, Arai H, Kuwaha S, Sohma H. [Cortical deafness due to left unilateral brain damage--a case report (author's transl)]. Rinsho Shinkeigaku. 1980 Sep;20(9):735-41. Japanese.**	Non-English language
**Barraquer-Bordas L, Peña-Casanova J, Pons-Irazábal L. Sordera central sin disturbios afásicos por lesión temporal bilateral [Central deafness without aphasic disorders due to bilateral temporal lesion]. Acta Neurol Latinoam. 1980;26(3):165-74. Spanish.**	Non-English language

**Parving A, Salomon G, Elberling C, Larsen B, Lassen NA. Middle Components of the Auditory Evoked Response in Bilateral Temporal Lobe Lesions: Report on a Patient with Auditory Agnosia. Scand Audiol. 1980 Jan 12;9(3):161–7.**	Non-CD auditory central dysfunction
**Goldstein MN, Brown M, Hollander J. Auditory agnosia and cortical deafness: analysis of a case with three-year followup. Brain Lang. 1975 Jul;2(3):324-32.**	Uncertainty about the etiology or extension of the lesions
**Jerger J, Lovering L, Wertz M. Auditory disorder following bilateral temporal lobe insult: report of a case. J Speech Hear Disord. 1972 Nov;37(4):523-35.**	Uncertainty about the etiology of the lesions
**Albert ML, Sparks R, Von Stockert T, Sax D. A case study of auditory agnosia: linguistic and non-linguistic processing. Cortex. 1972 Dec;8(4):427-43.**	Non-CD auditory central dysfunction
**Jerger J, Weikers NJ, Sharbrough FW 3rd, Jerger S. Bilateral lesions of the temporal lobe. A case study. Acta Otolaryngol Suppl. 1969;258:1-51.**	Uncertainty about the etiology of the lesions
**Spreen O, Benton AL, Fincham RW. Auditory agnosia without aphasia. Arch Neurol. 1965 Jul;13:84-92.**	Non-CD auditory central dysfunction
